# Coherent Phonons in van der Waals MoSe_2_/WSe_2_ Heterobilayers

**DOI:** 10.1021/acs.nanolett.3c02316

**Published:** 2023-08-21

**Authors:** Changxiu Li, Alexey V. Scherbakov, Pedro Soubelet, Anton K. Samusev, Claudia Ruppert, Nilanthy Balakrishnan, Vitalyi E. Gusev, Andreas V. Stier, Jonathan J. Finley, Manfred Bayer, Andrey V. Akimov

**Affiliations:** † Experimentelle Physik 2, Technische Universität Dortmund, Otto-Hahn-Str. 4a, 44227 Dortmund, Germany; ‡ Laboratoire d’Acoustique de l’Université du Mans (LAUM), UMR CNRS 6613, Institut d’Acoustique - Graduate School (IA-GS), Le Mans Université, 72085 Le Mans, France; § Walter Schottky Institut and TUM School of Natural Sciences, Technische Universität München, Am Coulombwall 4, 85748 Garching, Germany; ∥ School of Chemical and Physical Sciences, Keele University, Keele ST5 5BG, United Kingdom; ⊥ School of Physics and Astronomy, University of Nottingham, Nottingham NG7 2RD, United Kingdom

**Keywords:** coherent phonons, van der Waals nanolayers, indirect excitons, THz radiation, pump−probe spectroscopy

## Abstract

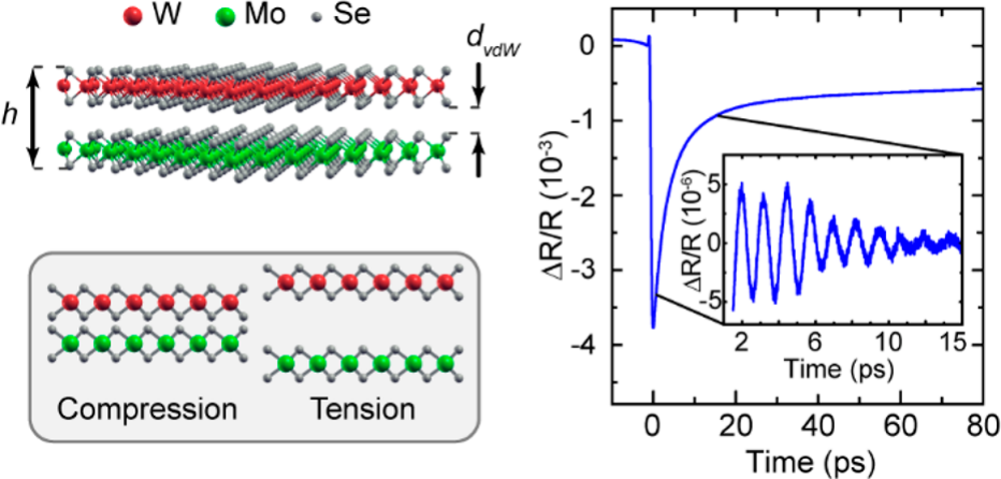

The increasing role of two-dimensional (2D) devices requires the development of new techniques for ultrafast control of physical properties in 2D van der Waals (vdW) nanolayers. A special feature of heterobilayers assembled from vdW monolayers is femtosecond separation of photoexcited electrons and holes between the neighboring layers, resulting in the formation of Coulomb force. Using laser pulses, we generate a 0.8 THz coherent breathing mode in MoSe_2_/WSe_2_ heterobilayers, which modulates the thickness of the heterobilayer and should modulate the photogenerated electric field in the vdW gap. While the phonon frequency and decay time are independent of the stacking angle between the MoSe_2_ and WSe_2_ monolayers, the amplitude decreases at intermediate angles, which is explained by a decrease in the photogenerated electric field between the layers. The modulation of the vdW gap by coherent phonons enables a new technology for the generation of THz radiation in 2D nanodevices with vdW heterobilayers.

The versatile “Lego-type” method for creating heterostructures by stacking, twisting, stretching, and bending two-dimensional (2D) monolayers provides a unique platform for manipulating optical, electrical, magnetic, piezoelectric, and spin properties in nanodevices.^1,2^ A challenge remains to control the properties of 2D heterostructures on an ultrafast time scale. To that end, coherent phonon technologies could become an efficient way to reach this goal, in a similar way to how coherent phonons have been used in traditional semiconductor nanostructures (e.g., quantum wells, nanolayers, superlattices, and nanocavities) to modulate optical,^3,4^ electronic^5,6^ magnetic,^7,8^ and plasmonic^9^ properties over ultrafast time scales. In 2D nanolayers, the dynamic strain associated with coherent phonons, depending on the phonon mode, either modulates the van der Waals (vdW) gap between monolayers (breathing mode) or produces an oscillating periodic bending or in-plane stretching (Lamb modes) on the nanometer scale. Strain-induced effects mediated by coherent phonons could pave a new way for ultrafast control in optoelectronic and THz devices.

Transition-metal dichalcogenide (TMD) homo- or heterobilayers are particularly interesting due to material and twist-dependent interlayer orbital couplings that fundamentally modify their electronic structure. Interlayer excitons are formed in TMD homo- and heterobilayers due to the spatial separation of photogenerated electrons and holes. Such excitations show a number of new physical phenomena, such as the coexistence of inter- and intralayer exciton species,^10,11^ DC Stark shift,^12,13^ exciton condensation,^14^ long-living spin polarization,^15^ and optically induced phase transitions^16^ as well as others (for a recent review see ref (17)). Excitons trapped in superlattice Moiré potential minima^18,19^ have been proposed to be useful for quantum information technologies,^20^ and the same lattice forms the basis for lattice-based photonic quantum simulators.^21^ The dynamic strain associated with coherent phonons modulates the electronic band structure in the vdW bilayers and affects various physical phenomena. Particularly, the generation of THz electromagnetic radiation as a result of distance modulation between charged monolayers could lead to a new paradigm, exploiting 2D vdW heterobilayers in THz technologies.

THz and sub-THz coherent phonons can be generated and detected by optical pulses.^22,23^ This technique has been used widely in experiments with relatively thick vdW layers and heterostructures.^24−30^ There are a few unique works where THz coherent phonons are studied in homobilayers fabricated from WSe_2_,^25^ MoSe_2_,^27^ and graphene,^30^ but to the best of our knowledge no coherent phonon experiments have been performed on heterobilayers. A specific physical feature for coherent phonon generation related to the Coulomb interaction between charged 2D monolayers is expected that distinguishes TMD heterobilayers from homobilayers and thicker vdW heterostructures. Indeed, electrons and holes generated optically in a heterobilayer become separated in real space: e.g. in MoSe_2_/WSe_2_ stacks, photogenerated electrons and holes become localized in MoSe_2_ and WSe_2_, respectively, on a time scale shorter than 100 fs.^17,31^ In addition to traditional mechanisms for ultrafast stress generation, e.g., thermoelastic and deformation potential,^22^ we thus expect the instant appearance of a Coulomb force which causes attraction of the layers, triggering THz coherent vibrations in the form of a resonant phonon breathing mode.

In the present Letter, we demonstrate the generation and detection of a coherent breathing mode in freely suspended MoSe_2_/WSe_2_ bilayers for a wide range of stacking angles ϑ. Using suspended layers is important to stop the escape of coherent phonons to the substrate.^26−29^ The results show that the measured frequency *f*_B_ ≈ 0.8 THz and decay time τ_B_ ≈ 5 ps of the coherent phonons are independent of ϑ. The analysis of optical detection and generation mechanisms shows that the ultrafast Coulomb attraction should be considered together with traditional thermoelastic and deformation potential mechanisms for coherent phonon generation.

The studied suspended heterobilayers are composed of mechanically exfoliated MoSe_2_ and WSe_2_ monolayers. Each monolayer consists of three 2D atomic layers, as shown in Figure 1a. The thickness of MoSe_2_ and WSe_2_ layers is approximately *d*_l_ ≈ 0.3 nm.^32^ The interlayer distance (i.e., the distance between the centers of the monolayers) in these 2D materials is known to be ∼0.7 nm.^33,34^ Then the width of the vdW gap in the heterobilayer is *d*_vdW_ ≈ 0.4 nm, and the total thickness of the heterobilayer is *h* = 2*d*_l_ + *d*_vdW_ ≈ 1 nm. The optical image of the flakes transferred on a patterned substrate with an array of holes with a 5 μm diameter is shown in Figure 1b. Room-temperature two-photon photoluminescence (PL) and second-harmonic-generation (SHG) experiments were performed to corroborate the number of layers and verify the stacking angle ϑ. Monolayers are identified through their A exciton emission,^35^ whose spectra are shown in Figure 1c. In addition to the two-photon PL emission, the spectra show the SHG signal. The angular distribution of the SHG intensity shown in Figure 1d allows us to obtain the stacking angle ϑ. Raman spectra of the monolayers and heterobilayer (see Figure 1e) show distinct peaks corresponding to the A_1g_ phonon modes in MoSe_2_ and WSe_2_ monolayers. Two sets of heterobilayers have been fabricated on two patterned substrates labeled as samples 1 and 2. In total, there are 6 and 2 heterobilayers on samples 1 and 2, respectively. For full details on the preparation and characterization of the heterobilayers, see Section 1 in the Supporting Information.

**Figure 1:**
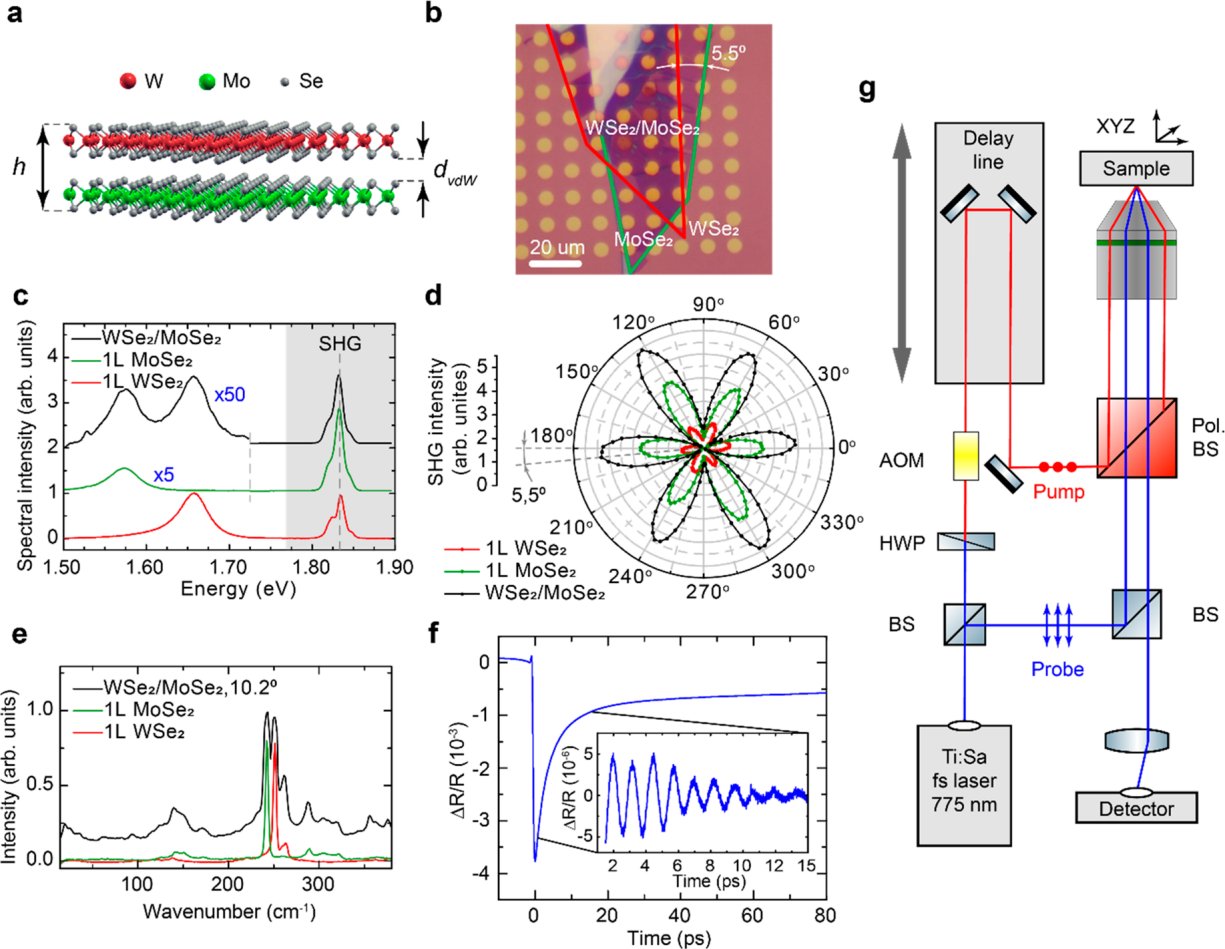
(a) Sketch of the MoSe_2_/WSe_2_ heterobilayer. (b) Microscope image of one sample stacked at 5.5° over the patterned holes. Green (red) lines indicate the MoSe_2_ (WSe_2_) monolayers. (c) Room-temperature (RT) two-photon photoluminescence (PL) and second-harmonic-generation (SHG) spectra recorded at different positions of the sample. Green and red spectra correspond to MoSe_2_ and WSe_2_ regions and display the characteristic RT single-layer PL. The bilayer region shows the emission from both layers. The strong PL quenching is consistent with the charge transfer between layers. (d) Polar plot of the polarization-resolved SHG intensity measured from each monolayer and the heterobilayer region. The relative angle between lobes as well as the relative intensity suggest a stacking angle of 5.5 ± 0.7°. (e) Raman spectra of monolayers and a heterobilayer. (f) Example of the pump–probe signal. The inset shows the signal after subtraction of slowly decaying background. (g) Pump–probe experimental setup: BS, beam splitter; HWP, half-wave plate; AOM, acousto-optical modulator.

A scheme of the pump–probe setup is shown in Figure 1g and an example of the measured transient reflectivity signal Δ*R*(*t*)/*R* is presented in Figure 1f (for details, see the Section 2 in the Supporting Information). There is a short leading edge with a duration of 0.9 ps and a recovery with a time constant of ∼10 ps. This signal is known to be related to the dynamics of photoexcited carriers, and this “electronic” signal will not be discussed further in the present work. The signal from coherent phonons excited in the heterobilayer by the pump pulse is superimposed on the background of the “electronic” signal and is shown in the inset of Figure 1f after subtraction of the background. Oscillatory behavior with an amplitude of Δ*R*(*t*)/*R* ≈ 10^–5^ due to coherent phonons is clearly observed.

Figure 2a,b shows background-free temporal signals Δ*R*(*t*)/*R* and their fast Fourier transforms (FFTs), respectively, measured on two sets of samples for various twist angles ϑ. All of the signals shown in Figure 2 are measured on the suspended layers. The signals for the bilayers on the substrate have higher noise level (Section 3 in the Supporting Information). The signals Δ*R*(*t*)/*R* are well described by the decaying oscillatory function . Correspondingly, the FFTs show a spectral line centered for both samples at *f*_B_ = 0.8 ± 0.03 THz. Spectral line shapes which depend on the phonon scattering rate are different for various heterobilayers. The dependence of *f*_B_, τ_B_, and amplitude Δ*R*_0_ on the stacking angle is shown in Figure 3a,b, respectively. No distinct dependence of *f*_B_ and τ_B_ on ϑ is revealed. The amplitude of the oscillations at *f*_B_ scales linearly with the pump fluence *J* (see Figure 3c) but decreases when the monolayers are misaligned (ϑ ≈ 20–30°) relative to the signals measured at close to 0 and 60°. It is seen from the comparison of the signal amplitude in Figure 2 and, despite the large error bar, in the angle dependence in Figure 3b.

**Figure 2:**
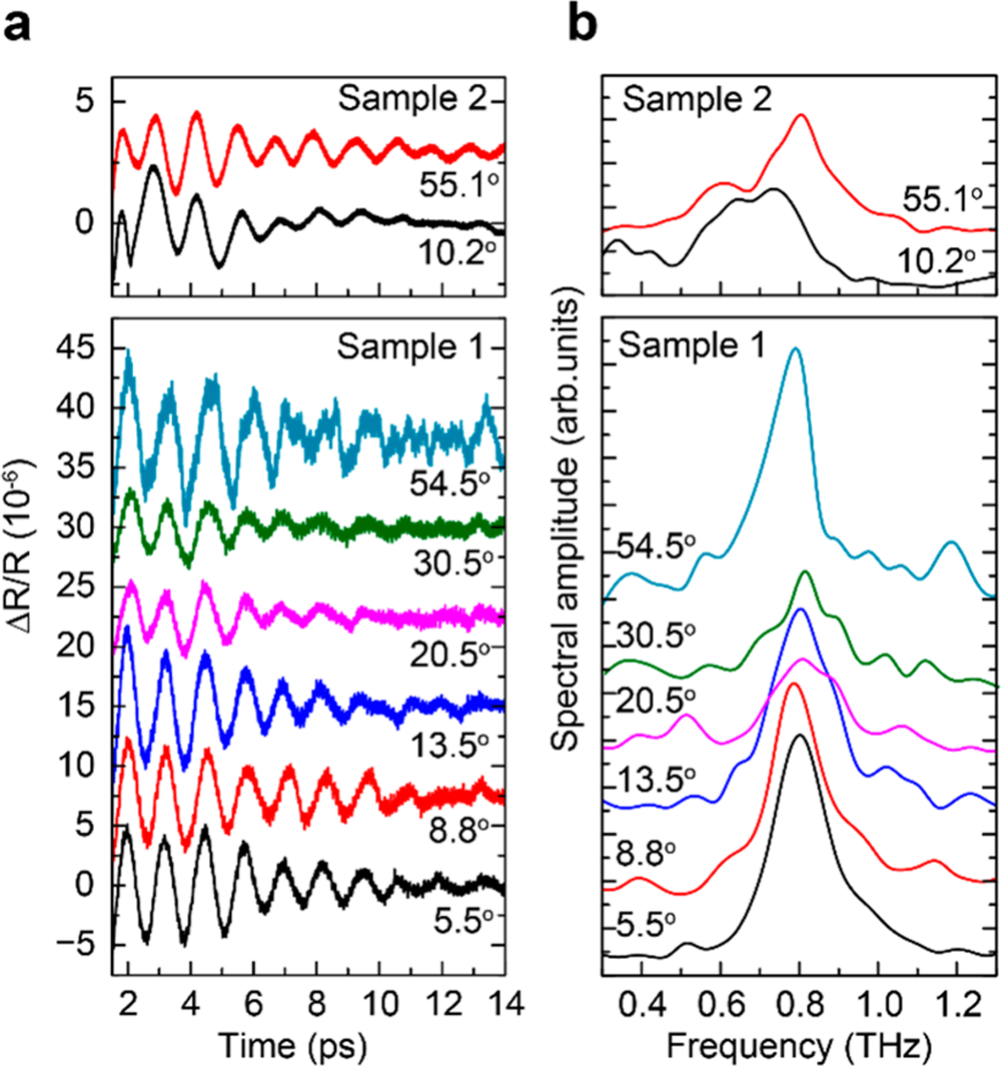
(a) Temporal signal Δ*R*/*R* after subtraction of the background measured in the flakes with various stacking angles. (b) Fast Fourier transforms (FFTs) of the signals shown in (a).

**Figure 3:**
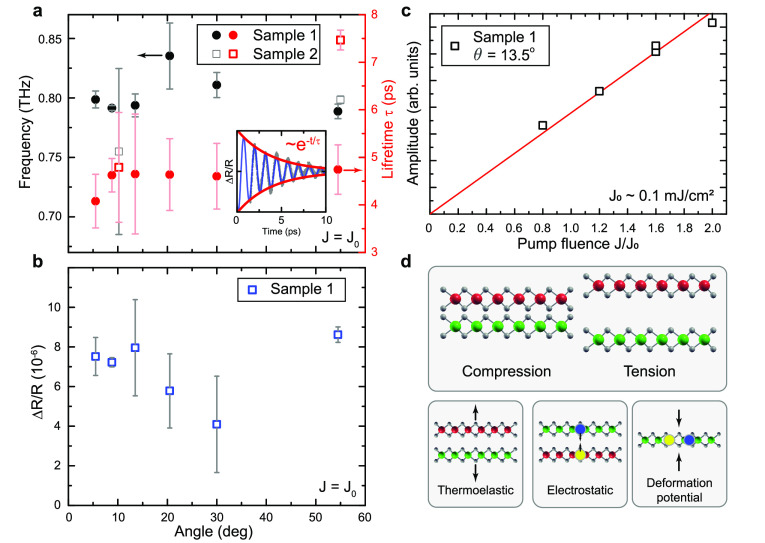
Dependences on the twist angle: (a) for frequency and lifetime; (b) reflectivity amplitude at pump fluence *J = J*_0_ ≈ 0.1 mJ/cm^2^. (c) Dependence of phonon amplitude on the pump fluence. The red line is the linear dependence. The inset in (a) is a demonstration of the fitting procedure for obtaining the lifetime and reflectivity amplitude of the phonon breathing mode. (d) Schematic illustration of layer displacement in breathing mode (top scheme) and schematics of three mechanisms for coherent phonon generation (bottom schemes).

The observation of coherent oscillations, whose frequency and decay time are independent of the stacking angle, is the main experimental result of our work. We attribute the oscillations in Δ*R*(*t*) to the coherent phonon breathing mode that corresponds to the modulation of the heterobilayer thickness *h*. Indeed, the measured frequency *f*_B_ = 0.8 ± 0.03 THz and decay time agree well with the values measured in Raman experiments in the same bilayers.^37^ Raman spectra measured on the studied bilayers (Section 4 in the Supporting Information) shows the line at the shift equal to *f*_B_ only when the backscattered and excitation beams have parallel polarization, which confirms that the origin of the spectral peak corresponds to the breathing mode. This result excludes the shear origin of the lines which should be present also in cross-polarization geometry, have essentially lower frequency and should be observed only for ϑ = 0° and ϑ = 60°.^37^ The independence of *f*_B_ on ϑ excludes the Moiré nature of the detected coherent phonons, which would show a strong dependence on ϑ,^38,39^ Our result is in good agreement with Raman experiments where no dependence of *f*_B_ on ϑ for the breathing mode in MoSe_2_/WSe_2_ heterobilayers was observed.^37^

The measured value of *f*_B_ allows us to calculate the stiffness *k*_vdW_ of the vdW elastic bond between MoSe_2_ and WSe_2_ monolayers using the equation
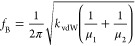
1where μ_1,2_ = 4.4 × 10^–6^ (5.9 × 10^–6^) kg m^–2^ is the sheet mass density for the MoSe_2_ (WSe_2_) monolayer. Substituting *f*_B_ = 0.8 ± 0.03 THz in (eq 1) we obtain *k*_vdW_ = (6.3 ± 0.4)×10^19^ N m^–3^, which agrees with a typical value for vdW nanolayers.^26,27,36^

The coherent breathing phonon mode was detected earlier in TMDs homobilayers of MoSe_2_^27^ and WSe_2_.^25^ The reported frequencies and decay times are  = 0.95 THz,  = 0.84 THz and  = 0.9 ps,  = 3.5 ps, respectively. We measure *f*_B_ ≈ 0.8 THz in MoSe_2_/WSe_2_ heterobilayers, which is slightly smaller than the frequency of the breathing mode in both homobilayers. This decrease could be explained by a smaller stiffness *k*_vdW_ of the vdW elastic bond in heterobilayers relative to homobilayers fabricated from the same materials. It is interesting that the decay time up to 7 ps measured in our experiments is longer than for the MoSe_2_ homobilayer^27^ and close to the value measured for the WSe_2_ homobilayer.^25^ It has been shown in ref (27) that the decay time in bilayers is due to the scattering at the interfaces and is much smaller than the anharmonic time of ∼100 ps for a frequency of ∼1 THz. The values of τ measured in our work support this conclusion and point at the dependence of phonon scattering on the growth method of the bulk material and the fabrication procedure, which to date cannot be controlled on the atomic level.

We continue by focusing the discussion on various coherent phonon detection and generation mechanisms. We start with the analysis of coherent phonon detection mechanisms. The dynamic strain associated with the phonon breathing mode results in the modulation Δ*h* of the heterobilayer thickness *h* and correspondingly to the modulation Δ*n̅* of the mean refractive index *n̅*. The detected optical signal Δ*R*(*t*)/*R* induced by the coherent phonons^40^ can be presented as the sum of two contributions. One is proportional to the modulation of the refractive index , and the other is proportional to the thickness modulation Δ*h*/*h*. Considering the real part of the monolayer permittivity ε_1_ = 25 (ε_1_ = 20)^41^ for MoSe_2_ (WSe_2_) at λ = 775 nm, we obtain a mean permittivity ε̅ = 13.9 and correspondingly *n̅* = 3.7 for the heterobilayer. The MoSe_2_ layer provides the main contribution to the photoelastic effect because the photon energy *E* = 2π*ℏc*/λ is in the vicinity of the direct exciton resonance in MoSe_2_,^41^ which is modulated by the dynamic strain associated with the coherent phonons. The exciton resonance in WSe_2_ lies at a higher *E* and does not contribute significantly to the photoelastic effect. Then for *h* ≪ λ, the equation for the amplitude of Δ*R*/*R* is (Section 5 in the Supporting Information)
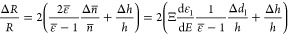
2where Ξ is the out-of-plane deformation potential for direct excitons in MoSe_2_, and Δ*d*_l_ corresponds to the thickness modulation of the MoSe_2_ layer by the coherent phonons. Theoretical calculations show that Ξ ∼ 1 eV,^42^ and at λ = 775 nm we estimate dε_1_/d*E* ≈ −10^2^ eV^–1^.^41^ Substituting these numerical values into (eq 2) we get
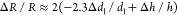
3

The sign of the calculated amplitude Δ*R*/*R* defines the phase of the oscillations, and in the case of several contributions that should be taken into account.

Now we turn to the phonon generation mechanisms. Optical pulses generate an instantaneous stress, which starts shifting the atoms of the heterobilayer and triggers a coherent phonon mode. The direction of atom shifting, i.e., compression or tension, may vary as shown in the schemes in Figure 3d. In the present work, we consider three mechanisms for phonon generation: electrostatic, thermoelastic, and deformation potential. The details for the last two may be found elsewhere.^22,43^

We first examine the *electrostatic mechanism*. The pump pulses with fluence *J* and wavelength λ = 775 nm excite electron–hole pairs with a sheet density , where *A* ≈ 1% is an absorbed fraction of pump light in MoSe_2_. The WSe_2_ monolayer is not excited directly by an optical pump due to very small absorption at 775 nm.^41^ In TMD heterobilayers the photoexcited carriers undergo ultrafast relaxation, and indirect excitons are formed over a time scale of ∼100 fs after electrons and holes become separated and localized in the MoSe_2_ and WSe_2_ monolayers, respectively.^17,31^ The ultrafast separation of the photoexcited carriers is supported by the strong (∼90%) PL quenching observed for the direct excitons in the heterobilayer relative to the PL in single MoSe_2_ and WSe_2_ monolayers (Figure 1c). The coupled electron–hole pairs cause an attractive Coulomb force *F*_c_ between the monolayers, which results in triggering the breathing mode of the heterobilayer. The Coulomb force on a unit area for the coupled electron–hole pairs, i.e., indirect excitons, may be written as
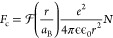
4where *r* = *d*_l_ + *d*_vdW_ is the interlayer distance equal to the distance between electron and hole in the bilayer^31^ and  is a form factor dependent on the ratio of exciton Bohr radius *a*_B_ and interlayer distance *r*. For *r* ≫ *a*_B_, 1. Considering *a*_B_ = 1.5 nm,^44^ we obtain  = 0.1.^45^ Using the value for the dielectric constant ϵ = 8,^46^ we estimate *F*_c_ ≈ (2.3 × 10^5^)*J* N m^–2^. Solving the elastic equation (Section 6 in the Supporting Information) we calculate the amplitudes Δ*h*/*h* = (−1.2 × 10^–6^*J*) and Δ*d*_l_/*d*_l_ = (−1.2 × 10^–7^)*J* where the excitation density *J* is in J m^–2^. Substituting these values into eq 3, we find Δ*R*_e_/*R* = (−1.8 × 10^–6^)*J*.

The second mechanism for stress generation is the *thermoelastic mechanism,* which is known to be the main mechanism in metals.^47^ The temperature rise from the ultrafast carrier relaxation may be written as  where *C*_1_ (*C*_2_) are the specific heat of the MoSe_2_ (WSe_2_) layers, *A* ≈ 1% is an absorbed fraction of pump light in MoSe_2_, and *B* = 0.14 is the fraction of energy converted to heat for carriers which undergo ultrafast hole relaxation from the MoSe_2_ to the WSe_2_ monolayers. Using *C*_1_ = 300 J (kg K)^−1^ ^48^ and *C*_2_ = 230 J (kg K)^−1^,^49^ we get Δ*T* ≈ 0.52*J*. We estimate the values for the thickness modulation as Δ*h*/*h* ≈ Δ*d*_l_/*d*_l_ = 0.5α_h_Δ*T*, where α_h_ ≈ α_d_ ≈ 10^–5^ are the out-of-plane expansion coefficients.^50^ Finally, using eq 3 for the thermoelastic mechanism, we calculate Δ*R*_t_/*R* = (−6.7 × 10^–6^)*J*.

Lastly, the *deformation potential mechanism* for stress generation is well-known for semiconductors.^43^ The photoexcitation of the MoSe_2_ layer induces stress given by σ_B_ = Ξ*N*/*d*. Due to the ultrafast carrier separation, the stress should appear in both monolayers but the values of Ξ for electrons and holes separately are not known. We shall assume that the effect of electrons and holes localized in different layers gives an effect similar to that for the direct excitons with Ξ = 1 eV localized in one layer. For the parameters used, we get σ_B_ = (2.1 × 10^7^)*J* N/m^2^. Then solving the elastic equation (Section 6 in the Supporting Information), we calculate the amplitudes for the breathing modes Δ*h*/*h* = (−2.5 × 10^–6^)*J* and Δ*d*_l_/*d*_l_ = (−2.5 × 10^–7^)*J*. From eq 3 we deduce Δ*R*_d_/*R* = (−3.9 × 10^–6^)*J*.

Adding all contributions together and substituting the pump fluence *J* ≈ 1 J m^–2^ used in the experiments, we get Δ*R*_Σ_/*R* = −1.2 × 10^–5^, which is slightly larger than what we observe in the experiment, |Δ*R*/*R*| ≈ 8 × 10^–6^ (see Figure 3b). However, we find this difference quite reasonable considering that parameters such as Ξ, ϵ, α_h_, and α_d_ used in the analysis are not well-known. All considered mechanisms result in a linear dependence of Δ*R*/*R* on *J* which is in agreement with the experiment (Figure 3c). We also considered other possible mechanisms (light pressure, piezo- and electrostriction), but their contribution is much smaller than those of the three considered above (Section 7 in the Supporting Information).

From the above estimates, we see that all contributions are at the same level of Δ*R*/*R* ≈ 10^–6^, indicating their joint importance in breathing mode generation. All mechanisms are known from earlier experiments: the deformation potential and thermoelastic mechanisms are most common in semiconductors,^22,43^ and the ultrafast separation of charges well-known for heterobilayers^17,31^ should evidently lead to the electrostatic impact. The importance of all three mechanisms follows from the fact that the calculated sum does not contradict the measured values.

The important role of the electrostatic mechanism follows from the dependence of the amplitude Δ*R*/*R* on the stacking angle, where Δ*R*/*R* possesses a minimum for ϑ∼30° as seen in Figure 3b. The thermoelastic and deformation potential mechanisms should not depend on ϑ so that the decrease in Δ*R*/*R* points to the decrease in *F*_c_ when the monolayers are misaligned. Such a statement is supported by the quenching of the indirect excitons in MoSe_2_/WSe_2_ heterobilayers, which is explained by the weakness of the interlayer coupling in the intermediate range of ϑ.^51^ A way to increase the role of the electrostatic contribution could be to perform experiments similar to those here at low temperatures, where the theory predicts formation of exciton–phonon polarons due to the interaction of indirect excitons with 2D flexural phonons.^45^

The analysis above shows that coherent phonon generation and detection in heterobilayers have specific features in comparison to those in homobilayers. The electrostatic and thermoelastic mechanisms are not typical for homobilayers, where no ultrafast separation or relaxation of photoexcited carriers take place. The experimental fact that in our experiments Δ*R*/*R* is higher than the maximum measured Δ*R*/*R* ≈ 10^–6^ in MoSe_2_ homobilayers^27^ supports the importance of all three phonon generation mechanisms in heterobilayers.

In conclusion, we have generated and detected coherent vibrations in MoSe_2_/WSe_2_ heterobilayers with a frequency of ∼0.8 THz and lifetimes of up to 5 ps. The measured frequency is independent of the stacking angle between the monolayers, which together with the agreement of the vibration frequency with the Raman data unambiguously allows us to attribute the vibrations to the breathing phonon mode, which is the only localized out-of-plane acoustic mode in the bilayer nanostructure. The analysis of phonon detection and generation mechanisms in heterobilayers shows a difference from homobilayers, where the detected signal is of smaller amplitude. Particularly, the electrostatic and thermoelastic mechanisms start playing a role in the phonon generation process due to the ultrafast carrier relaxation between the monolayers. The experimental demonstration of THz coherent phonons in heterobilayers paves the way to revealing new phenomena related to dynamic strain-induced effects. One of the most attractive applications would be phonon-induced modulation of the electric field from the electrons and holes localized in neighboring layers. Then, vdW heterobilayers excited by optical pulses may become the sources for THz microwave radiation. In more sophisticated 2D devices, such as p-n junctions^52^ or thermoelectric devices, coherent phonons could be used for generation of microwave currents as was done in traditional piezoelectric multilayer structures.^53^

## Supporting Information Available

Samples, pump–probe measurements, the difference between pump–probe signals for suspended bilayers and bilayers on the substrate, low-frequency Raman spectra, detection of coherent phonons, elastic equations, generation of breathing mode, electrostriction and light pressure (PDF)
