# Supplementary material for: Coherent Phonons in van der Waals MoSe2/WSe2 Heterobilayers
Source: Nano Lett. 2023 Aug 21;23(17):8186–93. doi: 10.1021/acs.nanolett.3c02316 (PMC10510584; doi:10.1021/acs.nanolett.3c02316)
Supplement: Supplementary file 1 — nl3c02316_si_001.pdf [file nl3c02316_si_001.pdf]

Coherent phonons in van der Waals $MoSe_2/WSe_2$ heterobilayers

Changxiu Li[1,2], Alexey V. Scherbakov[1], Pedro Soubelet[3], Anton K. Samusev[1], Claudia Ruppert[1], Nilanthy Balakrishnan[4], Vitalyi E. Gusev[2], Andreas V. Stier[3], Jonathan J. Finley[3], Manfred Bayer[1], Andrey V. Akimov[5]*.

[1]*Experimentelle Physik 2, Technische Universität Dortmund, Otto-Hahn-Str. 4a, 44227 Dortmund, Germany.*
[2]*Laboratoire d'Acoustique de l'Université du Mans (LAUM), UMR 6613, Institut d'Acoustique - Graduate School (IA-GS), CNRS, Le Mans Université, 72085 Le Mans, France.*
[3]*Walter Schottky Institut and TUM School of Natural Sciences, Technische Universität München, Am Coulombwall 4, 85748 Garching, Germany.*
[4]*School of Chemical and Physical Sciences, Keele University, Keele ST5 5BG, United Kingdom*
[5]*School of Physics and Astronomy, University of Nottingham, Nottingham NG7 2RD, United Kingdom*

* e-mail: andrey.akimov@nottingham.ac.uk

Supporting information

1. Samples

The flakes are exfoliated on separate viscoelastic stamps through the Scotch tape method. The crystallographic direction of different flakes is determined optically and considered during the stacking procedure to obtain a heterobilayer with pre-set stacking angle ϑ. Monolayers are deterministically transferred flake by flake [S1] to the pre-patterned substrate with 3 or 5 μm in diameter holes to obtain suspended TMDC heterobilayers. The layer number of the mechanically thinned 2D materials is determined by optical contrast. For PL and SHG, we use a 100-fs pulsed laser focused to a diffraction limited spot of 4 μm with a Cassegrain objective and a photon energy 2ω = 1.85 eV to probe the samples above the bandgap of each material. When performed on each layer and on the heterobilayer, we obtain the stacking angle considering that the SHG signal of each heterobilayer corresponds to the vectorial addition of the emission of each monolayer [S2]. Consequently: (i) the angle α between the SHG lobes of each layer provides the stacking angle with an indeterminacy of 60°, i.e., the stacking angle is $\vartheta = \alpha$ or $\vartheta = 60° - \alpha$, (ii) heterobilayers stacked close to 0° angle display an SHG signal whose intensity is approximately the addition of the SHG signals of each monolayer (constructive interference). However, heterobilayers stacked close to 60° angle display an SHG signal whose intensity is approximately the difference of the SHG signals of each monolayer (destructive interference) [S2]. Therefore, the stacking angle was fully determined by comparing the SHG relative intensities of each layer and the heterobilayer.

2. Pump-probe measurements

The optical pulses are generated by a MIRA laser (775 nm wavelength, 120 fs pulse duration, 76 MHz repetition rate). The pump beam is focused on a spot of 1.5 μm diameter and the maximum excitation density is J~0.1 mJ cm^{-2}. The probe beam is focused on a spot 1.5 times smaller than the pump spot and the probe excitation density does not exceed 0.1 mJ cm^{-2}. The temporal scan is realized by a mechanical delay line which controls the time delay t between the pump and probe pulses. The scanning interval of 15-mm, which corresponds to a time window of 100-ps, is scanned repeatedly with 30-60 averages, depending on the signal-to-noise ratio. The scan speed used in the experiments varied from 0.15 to 1.2 mm s^{-1}. The pump beam is modulated at 100 kHz with an acousto-optical

modulator (AOM). The intensity $R(t)$ of the probe beam reflected from the heterobilayer is measured with a 500-kHz bandwidth photodetector and filtered with a 10 MHz lock-in amplifier. The overall temporal resolution of the setup is ~100 fs and the minimum detectable amplitude above the noise level is $\Delta R/R$~3x10^{-7}.

3. The difference between pump-probe signals for suspended bilayers and bilayers on the substrate

Figure S1 shows the signals measured in suspended bilayers (black lines) and in the bilayers on the substrate (red lines). Panels (a) and (b) show temporal signals as measured and after the subtraction of the slow decaying background. Panel (c) shows the FFT spectra of the measured signals. It is seen that for the bilayer on the substrate, the signal shows oscillatory behaviour with the same frequency 0.8 THz as in the suspended layer. However, the signal-to-noise ratio is essentially worse for the layer on the substrate which makes it difficult to obtain the lifetime and the amplitude of the signal.

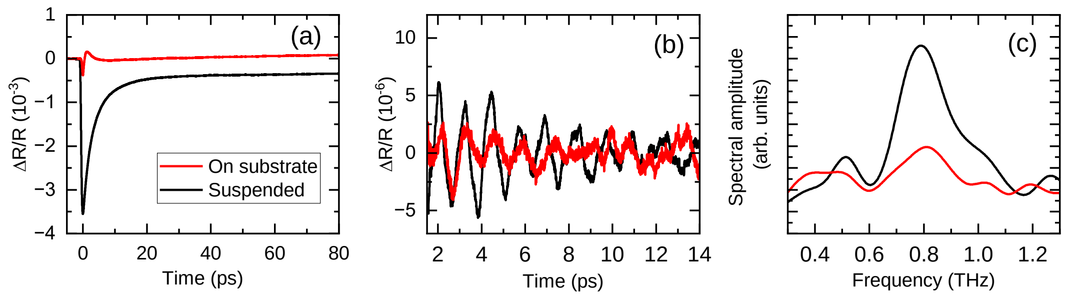

Figure S1. Signals for the same $MoSe_2/WSe_2$ bilayer (ϑ=13.5°) were measured at the suspended part (black lines) and on the part on the SiO_2/Si substrate. (a) temporal signals; (b) signals after the background subtraction; (c) Fast Fourier transforms of temporal signals shown in (b).

4. Low-frequency Raman spectra

Raman measurements were taken with a stabilized cw single mode laser at a wavelength of 531.52nm and the power on the sample was 11 µW. The Raman setup is based on two Ondax SureBlock ultra narrow-band notch filters, one of them also used for reflecting the laser radiation onto the sample to further reduce unwanted stray light from the laser. The laser was focused on the sample by a 100x microscope objective with a numerical aperture of 0.9 to a spot size of 0.6µm. The Raman signal was then detected in a KYMERA-328 spectrometer equipped with an Andor DU416A cooled CCD camera. The spectral resolution was about 3 cm^{-1} which corresponds to ~0.1 THz. Polarization resolved measurements were performed with a polarizer in front of the spectrometer. A lambda-half waveplate in front of the polarizer was used to switch between detecting radiation parallel (“parallel-polarized”) or perpendicular (“cross-polarized”) to the polarization of the exciting laser.

Figure S2(a) shows Raman spectra measure in $MoSe_2/WSe_2$ bilayer (ϑ=20.5°) for parallel and cross polarizations. It is seen that the mode at 0.8 THz is seen only in parallel polarization which confirms its origin as a breathing mode in a bilayer.

Figure S2(b) shows Raman spectra for bilayer (2L) and 3L-layer WSe_2. We present these spectra to be sure that the spectral peak at 0.8 THz (26.5 cm^{-1}) in (a) is related to the $MoSe_2/WSe_2$ bilayer. For 3L-WSe_2 the peak is obviously shifted to a lower frequency.

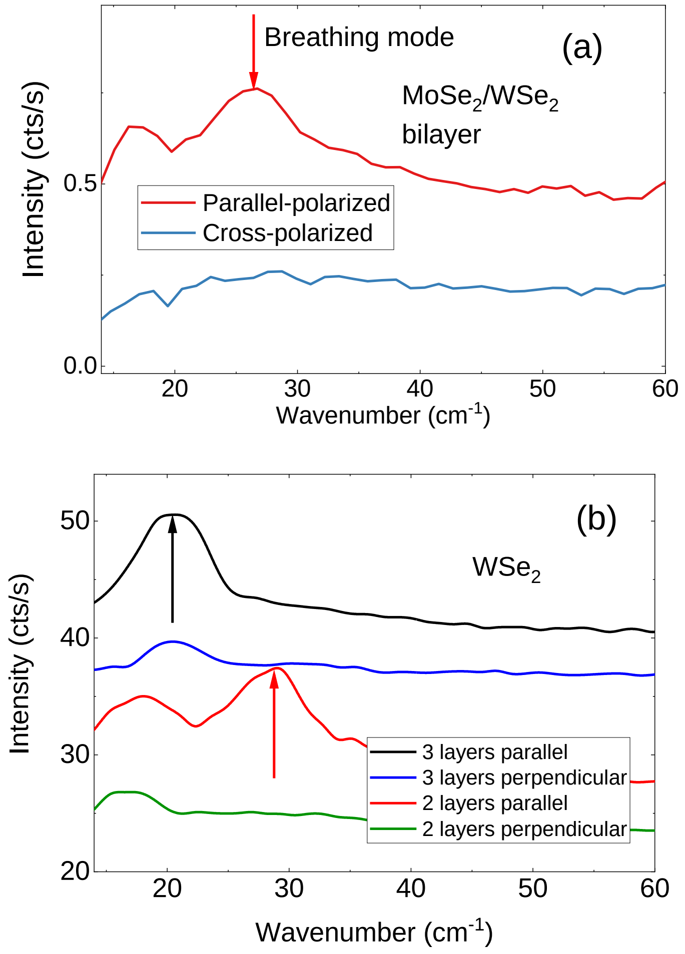

Figure S2. Low frequency Raman spectra in (a) $MoSe_2/WSe_2$ bilayer and (b) the layers of WSe_2 measured for parallel and crossed polarizations. The vertical arrows indicate the frequency of the breathing mode.

Figure S3 shows the low frequency Raman spectra of $MoSe_2/WSe_2$ bilayers with several stacking angles. It is clear that the frequency ~0.8 THz of the breathing mode does not depend on the stacking angle.

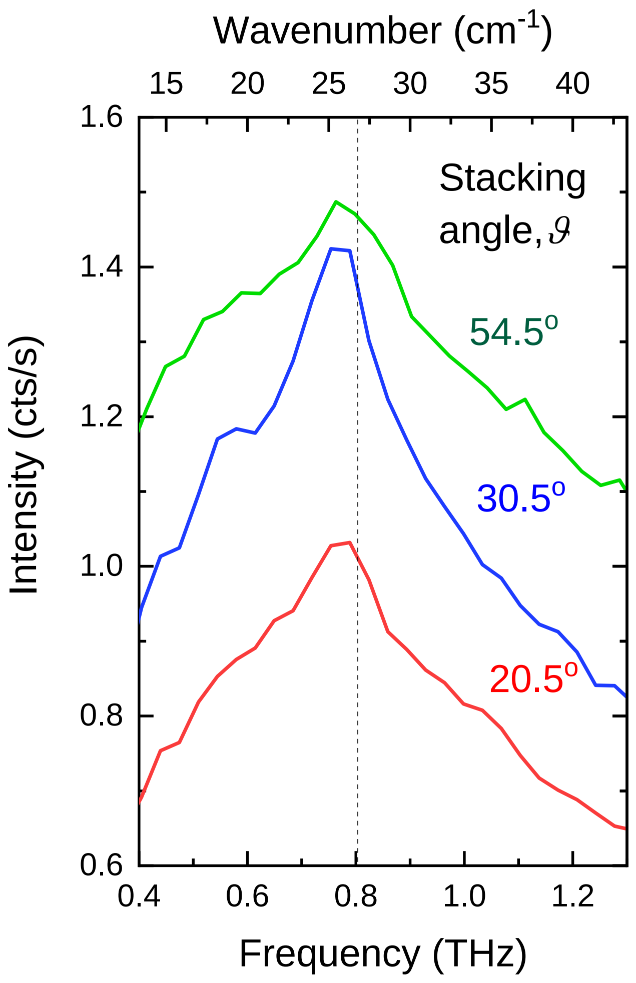

Figure S3. Raman spectra for $MoSe_2/WSe_2$ bilayers with different stacking angles.

5. Detection of coherent phonons

The reflectivity of light with wavelength λ from the layer with thickness h and mean refractive index $\bar{n}$ may be written as [S3]:

$$R = \frac{r_{12}^2 + r_{21}^2 + 2r_{12}r_{21}\cos 2\beta}{1 + r_{12}^2 r_{21}^2 + 2r_{12}r_{21}\cos 2\beta} \qquad \text{(S1)}$$

where $\beta = \frac{2\pi}{\lambda}\bar{n}h$, $r_{12} = \frac{1-\bar{n}}{1+\bar{n}} = r_0$ and $r_{21} = \frac{\bar{n}-1}{1+\bar{n}} = -r_0$. For thin layers when $\beta \ll 1$ we have

$$R = \left(\frac{2r_0}{1-r_0^2}\right)^2 \beta^2 \qquad \text{(S2)}$$

The dynamical strain associated with the coherent breathing mode results in changes of h and $\bar{n}$. As a result, the reflectivity changes by ΔR and differentiating Equation S2 for $\Delta R \ll R$ we get:

$$\Delta R = -\frac{8r_0\beta^2}{1-r_0^2}\frac{1+r_0^2}{\left(1-r_0^2\right)^2}\frac{2}{(1+\bar{n})^2}\Delta\bar{n} + 2\left(\frac{2r_0}{1-r_0^2}\right)^2 \beta\Delta\beta, \tag{S3}$$

and correspondingly

$$\frac{\Delta R}{R} = 2\left(\frac{2\bar{n}^2}{\bar{n}^2-1}\frac{\Delta\bar{n}}{\bar{n}} + \frac{\Delta h}{h}\right). \tag{S4}$$

The first term describes the photoelastic effect in the layer which is present only in the $MoSe_2$ monolayer due to the strong dependence of $\bar{n}$ on the photon energy E [S4].

We estimate the mean refractive index as:

$$\bar{n} = \sqrt{\bar{\varepsilon}} = \sqrt{\frac{d_1\varepsilon_1+d_2\varepsilon_2+d_g}{h}} \tag{S5}$$

where ε_1 and ε_2 are the permittivities of the $MoSe_2$ and WSe_2 monolayers respectively and the thicknesses of the layers $d_1 = d_2 = d_l$. Then for $\Delta\bar{n}$ induced by the strain η in the $MoSe_2$ layer associated from the thickness modulation we get:

$$\Delta\bar{n} = \frac{d\bar{n}}{d\eta}\eta = \frac{d\bar{n}}{d\varepsilon_1}\frac{d\varepsilon_1}{dE}\frac{dE}{d\eta}\eta\ . \tag{S6}$$

In vicinity of the exciton resonance $\frac{dE}{d\eta}$ is known as out of plane deformation potential Ξ for direct exciton and $= \frac{\Delta d_l}{d_l}$. From Equations S5 and S6 we get:

$$\frac{\Delta\bar{n}}{\bar{n}} = \Xi\frac{1}{2\sqrt{\bar{\varepsilon}}}\frac{d\varepsilon_1}{dE}\frac{d_l}{h}\frac{\Delta d_l}{d_l}\ . \tag{S7}$$

Finally, substituting S7 into S4 we get

$$\frac{\Delta R}{R} = 2\left(\Xi\frac{d\varepsilon_1}{dE}\frac{d_l}{h}\frac{1}{\bar{\varepsilon}-1}\frac{\Delta d_l}{d_l} + \frac{\Delta h}{h}\right). \tag{S8}$$

Equation S8 is identical to the Equation 2 in the main text.

6. Elastic equations

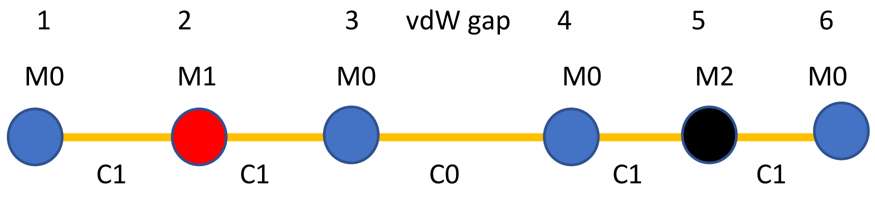

M_0- Se mass, *M_1*-Mo mass, *M_2*-W mass

F_{layer}- photoinduced stress in the layer, *F_{gap}* - photoinduced stress in the vdW gap

C_0- stiffness of vdW elastic bond; *C_1*- stiffness of elastic bond between the atoms in the layer

$$M_0\frac{d^2u_1}{dt^2} = C_1(u_2 - u_1) - F_{layer}$$
$$M_1\frac{d^2u_2}{dt^2} = C_1(u_1 - 2u_2 + u_3)$$
$$M_0\frac{d^2u_3}{dt^2} = C_1(u_2 - u_3) + C_0(u_4 - u_3) + F_{layer} - F_{gap}$$
$$M_0\frac{d^2u_4}{dt^2} = -C_0(u_4 - u_3) + C_1(u_5 - u_4) - F_{layer} + F_{gap}$$
$$M_2\frac{d^2u_5}{dt^2} = C_1(u_4 - 2u_5 + u_6)$$
$$M_0\frac{d^2u_6}{dt^2} = C_1(u_5 - u_6) + F_{layer}$$

The values for the elastic stiffnesses C_0 and C_1 are chosen to have an agreement with the frequencies for the breathing and A_{1g} mode respectively.

The equations are solved numerically using MATLAB software package.

7. Generation of breathing mode. Electrostriction and light pressure

In addition to thermoelastic, deformation potential and Coulomb interaction discussed in the main text, there are two additional mechanisms, that may contribute to the excitation of the breathing mode [S5]: *light pressure, inverse piezoelectric effect* and *electrostriction* mechanisms. In the present section we show that their contributions are negligible or absent in comparison with the three mechanisms discussed in the main text.

Light pressure on the individual layer, associated with the momentum transfer from light pulse to the material films, may be written as:

$$P = (A + 2R)\frac{I(t)}{c}, \tag{S9}$$

where A and R are absorbance and reflectance of the layer, $I(t)$ is the laser pulse intensity envelope and c is the light velocity in air. The term $\frac{I(t)}{c}$ is a rather small pressure by itself and the absorbance of the considered materials at the wavelength of our pump laser pulses is small (A of WSe_2 and of $MoSe_2$ are ~0.01 and ~0.025, respectively [S4]) as well as the reflectance (R of WSe_2 and of $MoSe_2$ are ~0.036 and ~0.038, respectively [S4]). Therefore, light pressures exhibited on the WSe_2 and $MoSe_2$ layers are only of about 4.6% and 6.3% of the characteristic light pressure $\frac{I(t)}{c}$. This explains the smallness of the light pressures in comparison with already considered stresses from electrostatic, deformation potential and thermoelastic mechanisms. The general intuition says that the observation of the light pressure effects is possible only if all other mechanisms are absent or forbidden by some reasons. In our system the light pressure effect is additionally reduced because the breathing mode could be driven only by the difference in the light pressures applied to our two layers.

Electrostriction stress $S_{ij}{\sim}a_{ijkl}E_kE_l$ may be induced in our layers due to the dependence of the dielectric tensor ϵ_{kl}, and, correspondingly, the electro-magnetic energy

density in the considered 2D materials on strain ($\partial\epsilon_{kl}/\partial\eta_{ij} \neq 0$, where η_{ij} is the strain tensor) and, thus, is directly related to photoelastic interaction. The second contribution to the electrostriction, in general, exists due to the dependence of the piezoelectric tensor e_{ijk}, on the electric field ($\partial e_{ijk}/\partial E_l \neq 0$). However, the components of the latter tensor, which could potentially contribute to excitation of breathing mode in our experimental geometry, are symmetry forbidden [S6]. On the one hand, this fact excludes an opportunity to excite the breathing mode via inverse piezoelectric effect by the THz electromagnetic pulse that could be generated in the layers by demodulation/rectification of our femtosecond pump laser pulse on the optical nonlinearity of the material. On the other hand, the tensor components $\frac{\partial e_{33k}}{\partial E_k}$, which could produce out-of-plane stresses via electrostriction effect [S6]. Here the x_3 is directed normally to the layers.

Due to nonzero tensor components $\partial\epsilon_{11}/\partial\eta_{33}$ we get a contribution

$(\frac{1}{2})\partial\epsilon_{11}/\partial\eta_{33}E_1E_1\eta_{33}$ to the electrostriction energy and finally to the photo-induced electrostrictive stress

$$S_{33} = \frac{1}{2}\frac{\partial\epsilon_{11}}{\partial\eta_{33}}E_1E_1. \tag{S10}$$

Here the x_1 coordinate is directed inside the layers along the electric field of the electric component of the pump laser. Photoelastic parameter $\partial\epsilon_{11}/\partial\eta_{33}$ can be estimated from the data on the refractive index, *n* [S7]:

$$S_{33} = \left(\frac{1}{2}\right)\left(\frac{\partial\epsilon_{11}}{\partial\eta_{33}}\right)E_1E_1 \approx \left(\frac{1}{2}\right)\epsilon_0\left(\frac{\partial\epsilon}{\partial\eta_{33}}\right)E^2 = \left(\frac{\partial\epsilon}{\partial\eta_{33}}\right)\frac{1}{nc}\left(\frac{n}{2c}E^2\right)=\left(\frac{\partial\epsilon}{\partial\eta_{33}}\right)\frac{I_n(t)}{nc}=$$

$$= \left(\frac{\partial\epsilon}{\partial E_g}\frac{\partial E_g}{\partial\eta_{33}}\right)\frac{I_n(t)}{nc} = \left(\frac{\partial\epsilon}{\partial E_g}\Xi\right)\frac{I_n(t)}{nc}, \tag{S11}$$

where $I_n(t)$ is the intensity of the electromagnetic wave in the material and E_g is the characteristic energy in the dependence of the dielectric function on optical quantum energy. In the studied heterobilayers for the used pump wavelength (775 nm) only $MoSe_2$ has a significant deformation potential $\Xi \equiv \dfrac{\partial E_g}{\partial\eta_{33}} \sim 1$ eV and exhibits large variation of the dielectric function $\frac{\partial\epsilon}{\partial E_g} \sim 100\ eV^{-1}$. Therefore, we consider electrostriction stress only in $MoSe_2$, where at our laser wavelength the real part of dielectric function largely dominates over the imaginary part [S4], i.e., $n = \sqrt{\epsilon}$ is real. This leads to the following relation between the laser intensity inside the layer $I_n(t)$ with the incident laser intensity:

$$I_n(t) = \frac{4n}{(1-n)^2}I(t). \tag{S12}$$

Then the electrostriction stress takes the final form for the estimates:

$$S_{33} = \left(\frac{\partial\epsilon}{\partial E_g}\Xi\right)\frac{4}{(1-n)^2}\frac{I(t)}{c}. \tag{S13}$$

Therefore, the electrostriction stress is proportional to the characteristic light pressure $\frac{I(t)}{c}$, with the proportionality constant, which for $MoSe_2$ ($n \cong 5$ for our optical wavelength) is much larger than 1, $\left(\frac{\partial e}{\partial E_g}\Xi\right)\frac{4}{(1-n)^2} \cong 25$. Thus, the electrostriction stress S_{33} is significantly larger than the characteristic light pressure given by Eq.(S9) and the action of the light pressure on our bi-layered structure is negligible in comparison with the action of the electrostriction stress.

We may estimate the value of the electrostriction stress in $MoSe_2$ from Eq.(S13) substituting $I(t) = \sqrt{\frac{8}{\pi}}\frac{J}{\tau_L}f(t)$, where τ_L is the full duration of the normalized Gaussian laser pulse intensity envelope $f(t)$ at the level 1/e^2 and *J* is the energy density of the pump laser pulse. The estimate results in

$$S_{33} \cong 10^6 Jf(t) \text{ N/m}^2, \qquad \text{(S14)}$$

where τ_L was assumed to be 130 fs and *J* is the energy of laser pulses measured in J/m^2.

For an accurate comparison of the electrostriction contribution to the breathing mode excitation with those from the deformation potential, it is important to take into account the difference in the temporal evolution of the respective stresses. For the assumed Gaussian $f(t) = exp\left[-\left(2\sqrt{2}t/\tau_L\right)^2\right]$, the normalized temporal rise of the deformation potential and thermoelastic stress is described by $\varphi(t) = \frac{1}{2}\left[1 + erf\left(2\sqrt{2}t/\tau_L\right)\right]$. The ratio of the Fourier spectra of these two dynamics can be evaluated as $\tilde{\varphi}(\omega)/\tilde{f}(\omega) = i\frac{2\sqrt{2}}{\sqrt{\pi}\omega\tau_L}$. At the frequency of the breathing mode, i.e. 0.8 THz, the modulus of this factor is 2.4. Finally, comparing spectral densities for the stress generated by electrostriction and deformation potential [$\sigma_B = -2.1 \times 10^7 J\varphi(t)$] we get that the second produces 50 times stronger stress than the first one. That is why in the manuscript we perform the strain estimations only for three mechanisms: electrostatic; thermoelastic; and deformation potential.

References

S1. Castellanos-Gomez, A.; Buscema, M.; Molenaar, R.; Singh, V.; Janssen, L.; van der Zant, H. S. J.; Steele, G. A. Deterministic Transfer of Two-Dimensional Materials by All-Dry Viscoelastic Stamping. *2D Mater.* **2014**, 1, 011002.

S2. Hsu, W. T.; Zhao, Z. A.; Li, L. J.; Chen, C. H.; Chiu, M. H.; Chang, P. S.; Chou, Y. C.; Chang, W. H. Second Harmonic Generation from Artificially Stacked Transition Metal Dichalcogenide Twisted Bilayers. *ACS Nano* **2014**, 8, 3, 2951–2958.

S3. Born, M., Wolf, E. Principles of optics; electromagnetic theory of propagation, interference and diffraction of light (2nd rev. ed.). New York: Pergamon Press. **1964**.

S4. Li, Y. L.; Chernikov, A.; Zhang, X.; Rigosi, A.; Hill, H. M.; van der Zande, A. M.; Chenet, D. A. ; Shih, E. M.; Hone, J.; Heinz, T. F. Measurement of the Optical Dielectric Function of Monolayer Transition-Metal Dichalcogenides: MoS_2, $MoSe_2$, WS_2, and WSe_2. *Phys. Rev. B* **2014**, 90, 205422.

S5. S. A. Akhmanov, S. A.; Gusev, V. E. Laser Excitation of Ultrashort Acoustic Pulses: New Advantages in Solid State Spectroscopy, the Investigation of Fast Processes and Nonlinear Acoustics. *Sov. Phys. Uspekhy* **1992**, 35, 3, 153-191; Ruello, P.; Gusev, V. Physical Mechanisms of Coherent Acoustic Phonons Generation by Ultrafast Laser Action. *Ultrasonics* **2015**, 56, 21-35.

S6. Duerloo, K. A. N.; Ong, M. T.; Reed, E. J. Intrinsic Piezoelectricity in Two-Dimensional Materials. *J. Phys. Chem. Lett.* **2012**, 3, 2871-2876.
